# Supplementary material for: Efficacy Studies against PCV-2 of a New Trivalent Vaccine including PCV-2a and PCV-2b Genotypes and Mycoplasma hyopneumoniae When Administered at 3 Weeks of Age
Source: Vaccines (Basel). 2022 Dec 9;10(12):2108. doi: 10.3390/vaccines10122108 (PMC9784864; doi:10.3390/vaccines10122108)
Supplement: Supplementary file 1 [file vaccines-10-02108-s001.zip › vaccines-2023567-supplementary.pdf]

Supplementary Materials:

Table S1. Number of animals enrolled per each action and timepoint performed in clinical studies.

**Weeks of age of study animals**	**Action performed**	**Num. of animals**			
		**Field trial A**		**Field trial B**	
		**Vaccinated pigs**	**Non-vaccinated pigs**	**Vaccinated pigs**	**Non-vaccinated pigs**
**3**	Body weight	328	335	392	394
	Blood sampling	50	52	51	51
**7**	Faecal swabs	48	51	48	49
	Blood sampling	48	51	48	49
**11**	Faecal swabs	47	49	39	39
	Blood sampling	47	49	39	39
**16**	Body weight	349	356	438	404
	Faecal swabs	45	46	59	62
	Blood sampling	45	46	58*	61*
**20**	Faecal swabs	47	47	57	58
	Blood sampling	47	47	58	59
**24-27**	Body weight	400	413	393	395
	Faecal swabs	45	46	57	58
	Blood sampling	45	46	57	58

The number of animals weighed and sampled were not the same at each timepoint due to deviations occurring during the study (death animals or animals not found at the moment).
*Blood sample from 2 animals were recorded wrong in study form and it is not possible to determine to which sample corresponds to which animal. Therefore, laboratory results corresponding to these two animals were excluded from analysis.

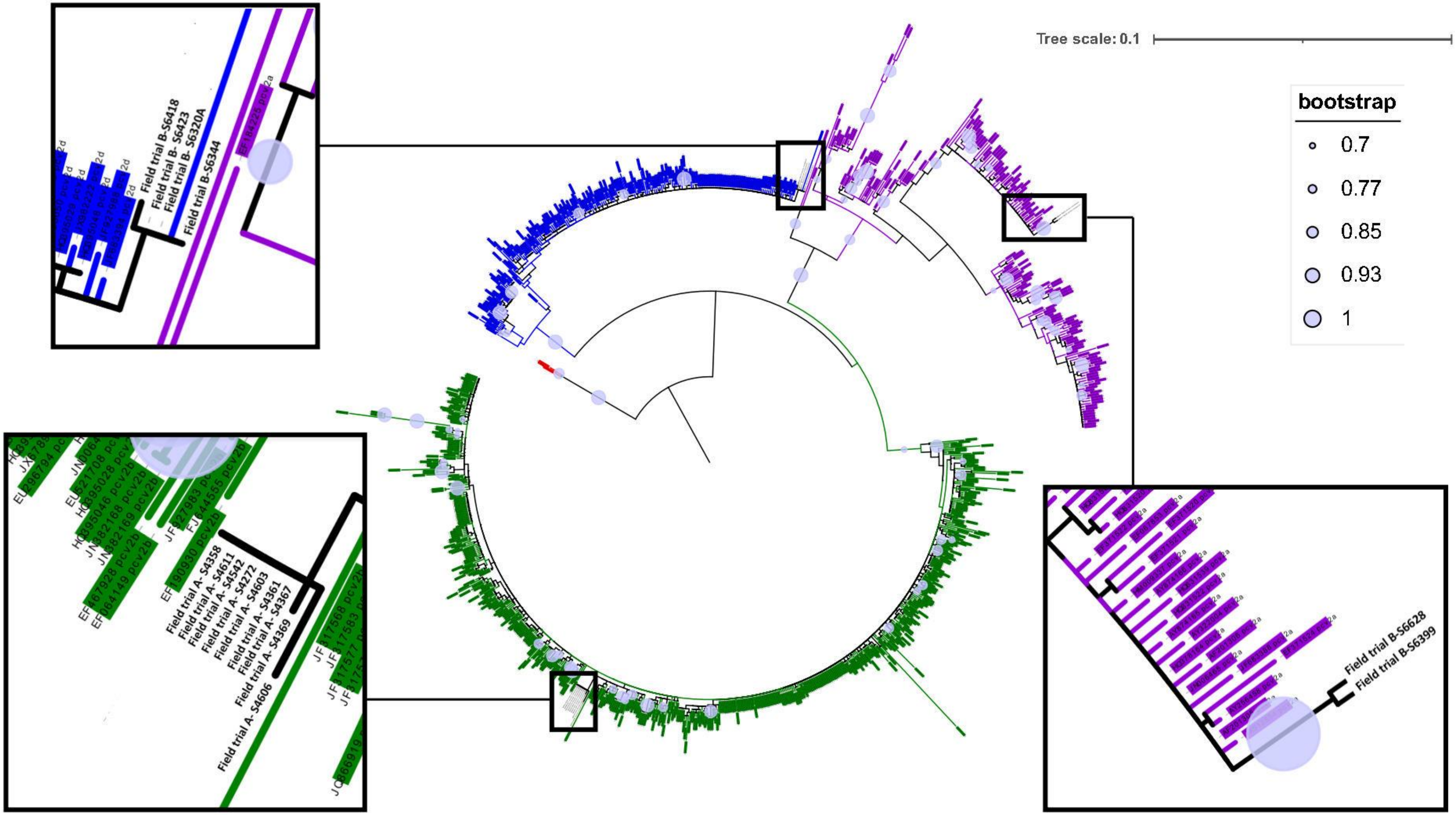

Figure S1: Phylogenetic tree derived from PCV-2 capsid protein (ORF2) sequences. Sequences from this study are indicated as Filed trial A or B plus the sample identification. The phylogenetic tree includes the relationships among the ORF2 sequences indicated with circles.
